# Quantum Correlation Swapping between Two Werner States Undergoing Local and Nonlocal Unitary Operations

**DOI:** 10.3390/e24091244

**Published:** 2022-09-04

**Authors:** Chuanmei Xie, Zhanjun Zhang, Jianlan Chen, Xiaofeng Yin

**Affiliations:** 1 School of Physics and Optoelectronic Engineering, Anhui University, Hefei 230039, China; 2 School of Information and Electronic Engineering, Zhejiang Gongshang University, Hangzhou 310018, China

**Keywords:** quantum correlation swapping, werner-like state, measurement-induced disturbance (MID), ameliorated MID (AMID), 03.65.Ta, 03.67.-a

## Abstract

In this paper, quantum correlation (QC) swapping between two Werner-like states, which are transformed from Werner states undergoing local and nonlocal unitary operations, are studied. Bell states measures are performed in the middle node to realize the QC swapping and correspondingly final correlated sates are obtained. Two different QC quantifiers, i.e., measurement-induced disturbance (MID) and ameliorated MID, are employed to characterize and quantify all the concerned QCs in the swapping process. All QCs in the concerned states are evaluated analytically and numerically. Correspondingly, their characteristics and properties are exposed in detail. It is exposed that, through the QC swapping process, one can obtain the long-distance QC indeed. Moreover, the similarities of monotony features of MID and AMID between the initial states and final states are exposed and analyzed.

## 1. Introduction

Quantum entanglement swapping is the core technique in quantum entanglement repeaters. Quantum entanglement repeaters are usually employed to realize long-distance quantum entanglements in some quantum tasks in quantum information processing [1,2,3,4,5,6,7]. Entanglement swapping can make a null-entanglement bipartite system entangled. Additionally, entanglement swapping can be utilized to enhance quantum entanglement [8].

Today, as is known to all, quantum correlations (QCs) no longer equal to quantum entanglement [9,10,11,12,13,14,15,16,17,18]. This recognition was exposed in 2001. Recently, Ollivier and Zurek [19] made a surprising discovery, in that there are indeed existing quantum correlation different from entanglement (QCDE). Later, numerous findings [20,21,22,23,24,25,26,27,28,29,30,31,32,33,34,35] about the recognition and applications of QCDEs appeared. Now, QCDE has become a hot field in the research of quantum information and computation.

As the quantum entanglement was generalized to quantum correlation, which can be quantum entanglement or QCDE, quantum entanglement swapping can also be generalized to quantum correlation swapping [36,37,38,39,40,41,42,43,44]. In quantum correlation swapping, the concerned quantum correlation may be quantum entanglement, QCDE, or both. Obviously, quantum correlation swapping is a general extentson of quantum entanglement swapping. Similarly, the realization of QCDE swapping can be in the same way with entanglement swapping. In many processes, the entanglement swapping and QCDE swapping can be realized simultaneously.

In the studies of quantum correlation swapping, three main aspects are of concern. One is the selection of initial states before QC swapping. Another is the selection of the middle measurement to realize the QC swapping. The last is the QC quantifiers, which are used to quantify the QCs in all the concerned states in the QC swapping process. Hence, one can see that complexities in the QC swapping stem from the above three aspects. Moreover, for the three aspects, one can see that many different selections can be used. Hence, many different properties can be exposed and revealed.

In this paper, a special QC swapping case will be considered. That is to say, two Werner-like states are taken as initial states; the four Bell state measurements are utilized to realize the QC swapping; and measurement-induced disturbance (MID) [20] and ameliorated MID (AMID) [23] are utilized to quantify the QCs in the concerned states.

The following is summarized for the rest of the paper. In Section 2, the Werner-like initial state QC swapping is described in detail. In Section 3, QCs in both the initial states and final states are quantified by MID. In Section 4, QCs in both the initial states and final states are quantified by AMID. In Section 5, QCs in the initial states, MID or AMID, are analyzed, discussed and compared. Lastly, in Section 6, a summary is provided.

## 2. Swapping QCs in Two Werner States Undergoing Local and Nonlocal Unitary Operations

In the QC swapping process, the two initial states are taken as a special kind of quantum-correlated states. It is called as Werner-like state because it is transformed from the famous Werner state undergoing local and nonlocal unitary operations.

Usually, a two-qubit Werner state can be written as

(1) $$\begin{array}{c} \varrho^{W}\left(z\right)=\frac{1-z}{4}I+z|\phi^{+}\rangle \langle \phi^{+}|, \end{array}$$

where I denotes a unit operator, $|\phi^{+}\rangle =(|00\rangle +|11\rangle )/\sqrt{2}$, *z* is real, and z∈(0,1]. When z≤1/3, Werner state $\varrho^{W}\left(z\right)$ is separable, while z>1/3, this state is entangled.

Through unitary operation U∈U(4), one can transform the Werner state $\varrho^{W}\left(z\right)$ to Werner-like state σ(z,c), i.e., $\sigma =U\varrho^{W}U^{+}$. Correspondingly, the Werner-like state $\sigma_{ab}\left(z,c\right)$ can be written as [44]

(2) $$\begin{array}{c} \sigma \left(z,c\right)=\frac{1-z}{4}I+z|\psi \rangle \langle \psi |, \end{array}$$

where $\left|\psi \rangle =U\right|\phi^{+}\rangle =\sqrt{c}|00\rangle +\sqrt{1-c}|11\rangle$ with c∈(0,1].

As for the QC swapping process in this study, the two initial Werner-like states are respectively written as

(3) $$\begin{array}{ccc} & & \sigma_{ab}\left(z_{1},c_{1}\right)=\frac{1-z_{1}}{4}I_{ab}+z_{1}|\psi_{1}\rangle_{ab}\langle \psi_{1}|, \end{array}$$

(4) $$\begin{array}{ccc} & & \sigma_{cd}\left(z_{2},c_{2}\right)=\frac{1-z_{2}}{4}I_{cd}+z_{2}|\psi_{2}\rangle_{cd}\langle \psi_{2}|, \end{array}$$

where $|\psi_{i}\rangle =\sqrt{c_{i}}|00\rangle +\sqrt{1-c_{i}}|11\rangle$, $z_{i},c_{i}\in \left(0,1\right]$ characterize the Werner-like states, and *I* is unit operator. *a*, *b*, *c* and *d* are four subsystems in the whole system, where *a* and *c* are located at a same place.

The QC swapping process can be described as follows. Alice has two particles *a* and *c*, Bob has a particle *b* and David has a particle *d*. Initially, *a* and *b* are in Werner-like state $\rho_{ab}$, while *c* and *d* are in Werner-like state $\rho_{cd}$. When Alice performs the middle measurement *a* and *c*, simultaneously, *b* and *d* will be in the final state $\rho_{bd}$. That is to say, initially, Bob and David have no any correlation. However, after the middle measurement performed by Alice, Bob and David will be correlated.

In the realization of QC swapping, the middle bipartite measurements are needed. In this paper, the following four qubit Bell states are selected as the middle bipartite measurements, i.e.,

(5) $$\begin{array}{c} {|\Phi \rangle }_{ac}^{\pm }=\frac{1}{\sqrt{2}}(|00\rangle \pm |11\rangle ), \end{array}$$

(6) $$\begin{array}{c} {|\Psi \rangle }_{ac}^{\pm }=\frac{1}{\sqrt{2}}(|01\rangle \pm |10\rangle ). \end{array}$$

By performing one of the middle measurements on the product states of Equations (3) and (4), a final state can be obtained. Corresponding to the four middle measurements, four final states appear. As for the two Bell states in Equation (5), the two final states can be obtained as the following:

(7) $$\begin{array}{ccc} \sigma_{bd}^{\pm }\left(z_{1},z_{2},c_{1},c_{2}\right) & = & \frac{1}{N}{\{W_{0}|0\rangle }_{b}{\langle 0||0\rangle }_{d}\langle 0|+W_{1}{|0\rangle }_{b}{\langle 0||1\rangle }_{d}\langle 1|+W_{2}{|1\rangle }_{b}{\langle 1||0\rangle }_{d}\langle 0|+W_{3}{|1\rangle }_{b}{\langle 1||1\rangle }_{d}\langle 1|]\\ & \pm & z_{1}z_{2}\sqrt{c_{1}c_{2}\left(1-c_{1}\right)\left(1-c_{2}\right)}{(|0\rangle }_{b}{\langle 1||0\rangle }_{d}\langle 1|+{|1\rangle }_{b}{\langle 0||1\rangle }_{d}\langle 0|)\}, \end{array}$$

where

(8) $$\begin{array}{c} N=W_{0}+W_{1}+W_{2}+W_{3}, \end{array}$$

and

(9) $$\begin{array}{ccc} & & W_{0}=\left(\frac{1-z_{1}}{4}+z_{1}c_{1}\right)\left(\frac{1-z_{2}}{4}+z_{2}c_{2}\right)+\frac{1-z_{1}}{4}\frac{1-z_{2}}{4},\\ & & W_{1}=\left(\frac{1-z_{1}}{4}+z_{1}c_{1}\right)\left[\frac{1-z_{2}}{4}\right]+\left[\frac{1-z_{2}}{4}+z_{2}\left(1-c_{2}\right)\right]\left(\frac{1-z_{1}}{4}\right),\\ & & W_{2}=\left(\frac{1-z_{2}}{4}+z_{2}c_{2}\right)\left(\frac{1-z_{1}}{4}\right)+\left[\frac{1-z_{1}}{4}+z_{1}\left(1-c_{1}\right)\right]\left[\frac{1-z_{2}}{4}\right],\\ & & W_{3}=[\left(\frac{1-z_{1}}{4}+z_{1}\left(1-c_{1}\right)\right]\left[\frac{1-z_{2}}{4}+z_{2}\left(1-c_{2}\right)\right]+\frac{1-z_{1}}{4}\frac{1-z_{2}}{4}. \end{array}$$

Note that the ± in Equation (7) corresponds to the middle measurements in Equation (5).

As for the two Bell states in Equation (6), the two final states can be obtained as the following:

(10) $$\begin{array}{ccc} \sigma_{bd}^{\prime \pm }\left(z_{1},z_{2},c_{1},c_{2}\right) & = & \frac{1}{N^{\prime }}{\{W_{0}^{\prime }|0\rangle }_{b}{\langle 0||0\rangle }_{d}\langle 0|+W_{1}^{\prime }{|0\rangle }_{b}{\langle 0||1\rangle }_{d}\langle 1|+W_{2}^{\prime }{|1\rangle }_{b}{\langle 1||0\rangle }_{d}\langle 0|+W_{3}^{\prime }{|1\rangle }_{b}{\langle 1||1\rangle }_{d}\langle 1|]\\ & \pm & z_{1}z_{2}\sqrt{c_{1}c_{2}\left(1-c_{1}\right)\left(1-c_{2}\right)}{(|0\rangle }_{b}{\langle 1||0\rangle }_{d}\langle 1|+{|1\rangle }_{b}{\langle 0||1\rangle }_{d}\langle 0|)\}, \end{array}$$

where

(11) $$\begin{array}{c} N^{\prime }=W_{0}^{\prime }+W_{1}^{\prime }+W_{2}^{\prime }+W_{3}^{\prime }, \end{array}$$

and

(12) $$\begin{array}{ccc} & & W_{0}^{\prime }=\left(\frac{1-z_{1}}{4}+z_{1}c_{1}\right)\frac{1-z_{2}}{4}+\left(\frac{1-z_{2}}{4}+z_{2}c_{2}\right)\frac{1-z_{1}}{4},\\ & & W_{1}^{\prime }=\left(\frac{1-z_{1}}{4}+z_{1}c_{1}\right)\left[\frac{1-z_{2}}{4}+z_{2}\left(1-c_{2}\right)\right]+\left(\frac{1-z_{1}}{4}\right)\left(\frac{1-z_{2}}{4}\right),\\ & & W_{2}^{\prime }=\left(\frac{1-z_{2}}{4}+z_{2}c_{2}\right)\left[\frac{1-z_{1}}{4}+z_{1}\left(1-c_{1}\right)\right]+\left(\frac{1-z_{1}}{4}\right)\left(\frac{1-z_{2}}{4}\right),\\ & & W_{3}^{\prime }=[\left(\frac{1-z_{1}}{4}+z_{1}\left(1-c_{1}\right)\right]\frac{1-z_{2}}{4}+\left[\frac{1-z_{2}}{4}+z_{2}\left(1-c_{2}\right)\right]\frac{1-z_{1}}{4}. \end{array}$$

Here in Equation (10), the ± correspond to the middle measurements in Equation (6).

## 3. MID in the Concerned States

Measurement-induced disturbance (MID) is a QC measure [22] that has been attracting considerable attention for its easy computability. MID is defined as the difference between the total correlation and its classical correlation, where, for a given concerned state, the classical correlation is determined by measuring both subsystems with the eigenvectors of marginal states as the measuring bases.

### 3.1. MIDs in the Two Initial Werner-Like States

For the two initial Werner-like states $\sigma_{ab}\left(z_{1},c_{1}\right)$ and $\sigma_{cd}\left(z_{2},c_{2}\right)$, their MIDs [45] are

(13) $$\begin{array}{ccc} Q_{M}\left[\sigma_{ab}\left(z_{1},c_{1}\right)\right] & = & -\left(\frac{1-z_{1}}{4}+z_{1}c_{1}\right)\log_{2}\left(\frac{1-z_{1}}{4}+z_{1}c_{1}\right)-\left(\frac{1+3z_{1}}{4}-z_{1}c_{1}\right)\log_{2}\left(\frac{1+3z_{1}}{4}-z_{1}c_{1}\right)\\ & & +\left(\frac{1-z_{1}}{4}\right)\log_{2}\left(\frac{1-z_{1}}{4}\right)+\left(\frac{1+3z_{1}}{4}\right)\log_{2}\left(\frac{1+3z_{1}}{4}\right), \end{array}$$

(14) $$\begin{array}{ccc} Q_{M}\left[\sigma_{cd}\left(z_{2},c_{2}\right)\right] & = & -\left(\frac{1-z_{2}}{4}+z_{2}c_{2}\right)\log_{2}\left(\frac{1-z_{2}}{4}+z_{2}c_{2}\right)-\left(\frac{1+3z_{2}}{4}-z_{2}c_{2}\right)\log_{2}\left(\frac{1+3z_{2}}{4}-z_{2}c_{2}\right)\\ & & +\left(\frac{1-z_{2}}{4}\right)\log_{2}\left(\frac{1-z_{2}}{4}\right)+\left(\frac{1+3z_{2}}{4}\right)\log_{2}\left(\frac{1+3z_{2}}{4}\right). \end{array}$$

### 3.2. MIDs in the Final States $\sigma_{bd}^{\pm }\left(z_{1},z_{2},c_{1},c_{2}\right)$

Now let us inspect the final state $\sigma_{bd}^{\pm }$ in Equation (7). Obviously, $\sigma_{bd}^{+}$ and $\sigma_{bd}^{-}$ are different. However, the difference is minor and it is located at the position ±. In the following calculations, one can find that the MID in $\sigma_{bd}^{+}$ is equivalent to that in $\sigma_{bd}^{-}$. That it to say, in the calculation of MIDs, the position + or − can be ignored. Hence, for convenience, in the following, $\sigma_{bd}^{\pm }$ can be obtained by $\sigma_{bd}$.

In the final state $\sigma_{bd}\left(z_{1},z_{2},c_{1},c_{2}\right)$, the total correlation can be obtained as the following

(15) $$I\left[\sigma_{bd}\left(z_{1},z_{2},c_{1},c_{2}\right)\right]=S\left[\sigma_{b}\left(z_{1},z_{2},c_{1},c_{2}\right)\right]+S\left[\sigma_{d}\left(z_{1},z_{2},c_{1},c_{2}\right)\right]-S\left[\sigma_{bd}\left(z_{1},z_{2},c_{1},c_{2}\right)\right],$$

where S[·] is von Neumann entropy, $\sigma_{b}\left(z_{1},z_{2},c_{1},c_{2}\right)$ and $\sigma_{d}\left(z_{1},z_{2},c_{1},c_{2}\right)$ represent marginal states of $\sigma_{bd}\left(z_{1},z_{2},c_{1},c_{2}\right)$ which take the form as

(16) $$\begin{array}{c} \left.\begin{array}{c} \sigma_{b}\left(x,y,\kappa \right)=\frac{1}{4}{[\left(w_{0}+w_{1}\right)|0\rangle }_{b}\langle 0|+\left(w_{2}+w_{3}\right){|1\rangle }_{b}\langle 1|],\\ \sigma_{d}\left(x,y,\kappa \right)=\frac{1}{4}{[\left(w_{0}+w_{2}\right)|0\rangle }_{d}\langle 0|+\left(w_{1}+w_{3}\right){|1\rangle }_{d}\langle 1|], \end{array}\right\} \end{array}$$

where $w_{0}=\frac{4}{N}W_{0}$, $w_{3}=\frac{4}{N}W_{3}$, $w_{1}=\frac{4}{N}W_{1}$, $w_{2}=\frac{4}{N}W_{2}$, and $W_{i}$’s are functions of $z_{1}$, $z_{2}$, $c_{1}$ and $c_{2}$ given by Equation (9). Easily, one can obtain

(17) $$\begin{array}{ccc} S[\sigma_{b}\left(z_{1},z_{2},c_{1},c_{2}\right)] & = & \frac{1}{4}\left[8-w_{01}\log_{2}w_{01}-w_{23}\log_{2}w_{23}\right], \end{array}$$

(18) $$\begin{array}{ccc} S[\sigma_{d}\left(z_{1},z_{2},c_{1},c_{2}\right)] & = & \frac{1}{4}\left[8-w_{02}\log_{2}w_{02}-w_{13}\log_{2}w_{13}\right], \end{array}$$

(19) $$\begin{array}{ccc} S[\sigma_{bd}\left(z_{1},z_{2},c_{1},c_{2}\right)] & = & -\frac{1}{4}\left[w_{1}\log_{2}w_{1}+w_{2}\log_{2}w_{2}-2w_{12}-3w_{03}\right]\\ & & -\frac{1}{8}\left[\left(w_{03}+\xi \right)\log_{2}\left(w_{03}+\xi \right)+\left(w_{03}-\xi \right)\log_{2}\left(w_{03}-\xi \right)\right], \end{array}$$

where $w_{mn}=w_{m}+w_{n}$ and $\xi =\sqrt{{\left(w_{3}-w_{0}\right)}^{2}+16\zeta }$ with $\zeta =4c_{1}\left(1-c_{1}\right)c_{2}\left(1-c_{2}\right)z_{1}^{2}z_{2}^{2}/N^{2}$.

To calculate MID in the final state $\sigma_{bd}\left(z_{1},z_{2},c_{1},c_{2}\right)$, its marginal states are needed. It is because that the eigenvectors of marginal states are taken as the measuring bases to aquire the classical correlation. Using the marginal states in Equation (16) as measuring bases to measure both subsystems simultaneously, four different outcomes can be obtained. For each outcome, its own probability may be occured. Let $p_{bd}^{\left(ij\right)}$ denote its occurrence probability where ${|ij\rangle }_{bd}$ is the corresponding outcome. It is easy to work out the occurrence probability as

(20) $$\begin{array}{c} p_{bd}^{\left(00\right)}=\frac{1}{4}w_{0},\;\;p_{bd}^{\left(01\right)}=\frac{1}{4}w_{1},\;\;p_{bd}^{\left(10\right)}=\frac{1}{4}w_{2},\;\;p_{bd}^{\left(11\right)}=\frac{1}{4}w_{3}. \end{array}$$

Integrating above probabilities, the single-partite probability distributions can be obtained:

(21) $$\begin{array}{c} \left.\begin{array}{ccc} p_{b}^{\left(0\right)}=p_{bd}^{\left(00\right)}+p_{bd}^{\left(01\right)}=\frac{1}{4}w_{01}, & & p_{b}^{\left(1\right)}=p_{bd}^{\left(10\right)}+p_{bd}^{\left(11\right)}=\frac{1}{4}w_{23},\\ p_{d}^{\left(0\right)}=p_{bd}^{\left(00\right)}+p_{bd}^{\left(10\right)}=\frac{1}{4}w_{02}, & & p_{d}^{\left(1\right)}=p_{bd}^{\left(01\right)}+p_{bd}^{\left(11\right)}=\frac{1}{4}w_{13}. \end{array}\right\} \end{array}$$

Utilizing Equations (20) and (21), the classical correlation in the final state $\sigma_{bd}\left(z_{1},z_{2},c_{1},c_{2}\right)$ can be obtained, i.e.,

(22) $$\begin{array}{ccc} C_{M}\left[\sigma_{bd}\left(z_{1},z_{2},c_{1},c_{2}\right)\right] & = & \frac{1}{4}\left(w_{0}\log_{2}w_{0}+w_{1}\log_{2}w_{1}+w_{2}\log_{2}w_{2}+w_{3}\log_{2}w_{3}\right)+2\\ & - & \frac{1}{4}\left(w_{01}\log_{2}w_{01}+w_{02}\log_{2}w_{02}+w_{13}\log_{2}w_{13}+w_{23}\log_{2}w_{23}\right), \end{array}$$

Finally, MID in the final state $\sigma_{bd}\left(z_{1},z_{2},c_{1},c_{2}\right)$ can be extracted as

(23) $$\begin{array}{ccc} Q_{M}\left[\sigma_{bd}\left(z_{1},z_{2},c_{1},c_{2}\right)\right] & = & I\left[\sigma_{bd}\left(z_{1},z_{2},c_{1},c_{2}\right)\right]-C_{M}\left[\sigma_{bd}\left(z_{1},z_{2},c_{1},c_{2}\right)\right]\\ & = & [\left(w_{03}+\xi \right)\log_{2}\left(w_{03}+\xi \right)+\left(w_{03}-\xi \right)\log_{2}\left(w_{03}-\xi \right)\\ & - & 2w_{0}\log_{2}w_{0}-2w_{3}\log_{2}w_{3}-2w_{03}]/8. \end{array}$$

### 3.3. MID in the Final States $\sigma_{bd}^{\prime \pm }\left(z_{1},z_{2},c_{1},c_{2}\right)$

From Equation (10), it is easy to find the difference between $\sigma_{bd}^{\prime +}\left(z_{1},z_{2},c_{1},c_{2}\right)$ and $\sigma_{bd}^{\prime -}\left(z_{1},z_{2},c_{1},c_{2}\right)$. It is + or −. Similar to that in Section 3.2, MIDs in the two final states are equivalent, i.e., MID in $\sigma_{bd}^{\prime +}\left(z_{1},z_{2},c_{1},c_{2}\right)$ is equivalent with that in $\sigma_{bd}^{\prime -}\left(z_{1},z_{2},c_{1},c_{2}\right)$. Hence, for convenience in the context, $\sigma_{bd}^{\prime }\left(z_{1},z_{2},c_{1},c_{2}\right)$ is considered instead.

Morovere, compare $\sigma_{bd}^{\prime \pm }\left(z_{1},z_{2},c_{1},c_{2}\right)$ in Equation (10) with $\sigma_{bd}^{\pm }\left(z_{1},z_{2},c_{1},c_{2}\right)$ in Equation (7), one can find that the two kinds of states have similar structure, only parameters in them are different. Hence, according to this similarity and the obtained MID of $\sigma_{bd}\left(z_{1},z_{2},c_{1},c_{2}\right)$ in Equation (22), one can directly accquire MID in $\sigma_{bd}^{\prime }\left(z_{1},z_{2},c_{1},c_{2}\right)$ as the following

(24) $$\begin{array}{ccc} Q_{M}\left[\sigma_{bd}^{\prime }\left(z_{1},z_{2},c_{1},c_{2}\right)\right] & = & [\left(w_{03}^{\prime }+\xi^{\prime }\right)\log_{2}\left(w_{03}^{\prime }+\xi^{\prime }\right)+\left(w_{03}^{\prime }-\xi^{\prime }\right)\log_{2}\left(w_{03}^{\prime }-\xi^{\prime }\right)\\ & - & 2w_{0}^{\prime }\log_{2}w_{0}^{\prime }-2w_{3}^{\prime }\log_{2}w_{3}^{\prime }-2w_{03}^{\prime }]/8, \end{array}$$

where all the $w^{\prime }$ are quantities related to those in Equation (23) with W’s are replaced by W′’s. W’s and W′’s are listed in Equations (9) and (12), respectively.

## 4. AMID in the Concerned States

Another QC measure, ameliorated measurement-induced disturbance (AMID), was put forward in 2011, in which the corresponding maximal classical correlation is special. The special aspect is that, to find the maximal classical correlation, optimization procedure to rehearse all joint local measurements is needed. Correspondingly, AMID is defined as the discrepancy between total correlation and the obtained maximal classical correlation.

### 4.1. AMID in the Two Werner-Like Initial States

For the two initial Werner-like states $\sigma_{ab}\left(z_{1},c_{1}\right)$ and $\sigma_{cd}\left(z_{2},c_{2}\right)$, their AMIDs [45] are

(25) $$\begin{array}{ccc} Q_{A}\left[\sigma_{ab}\left(z_{1},c_{1}\right)\right] & = & -\left(\frac{1-z_{1}}{4}+z_{1}c_{1}\right)\log_{2}\left(\frac{1-z_{1}}{4}+z_{1}c_{1}\right)-\left(\frac{1+3z_{1}}{4}-z_{1}c_{1}\right)\log_{2}\left(\frac{1+3z_{1}}{4}-z_{1}c_{1}\right)\\ & & +\left(\frac{1-z_{1}}{4}\right)\log_{2}\left(\frac{1-z_{1}}{4}\right)+\left(\frac{1+3z_{1}}{4}\right)\log_{2}\left(\frac{1+3z_{1}}{4}\right), \end{array}$$

(26) $$\begin{array}{ccc} Q_{A}\left[\sigma_{cd}\left(z_{2},c_{2}\right)\right] & = & -\left(\frac{1-z_{2}}{4}+z_{2}c_{2}\right)\log_{2}\left(\frac{1-z_{2}}{4}+z_{2}c_{2}\right)-\left(\frac{1+3z_{2}}{4}-z_{2}c_{2}\right)\log_{2}\left(\frac{1+3z_{2}}{4}-z_{2}c_{2}\right)\\ & & +\left(\frac{1-z_{2}}{4}\right)\log_{2}\left(\frac{1-z_{2}}{4}\right)+\left(\frac{1+3z_{2}}{4}\right)\log_{2}\left(\frac{1+3z_{2}}{4}\right). \end{array}$$

### 4.2. AMIDs in the Final States $\sigma_{bd}^{\pm }\left(z_{1},z_{2},c_{1},c_{2}\right)$

In order to evaluate AMID in $\sigma_{bd}^{\pm }\left(z_{1},z_{2},c_{1},c_{2}\right)$, a general joint local measurement should first be parameterized. It can be parameterized as $\{\Omega_{b}^{\left(i\right)}\left(\alpha_{1},\phi_{1},\tau_{1}\right)⊗\Lambda_{d}^{\left(j\right)}\left(\alpha_{2},\phi_{2},\tau_{2}\right),$i,j=0,1}, where $\Omega^{\left(k\right)}$ and $\Lambda^{\left(k\right)}$ take the same forms as that of $\Pi^{\left(k\right)}$ described as the following three-parameter forms:

(27) $$\begin{array}{c} \{\Pi^{\left(0\right)}\left(\alpha ,\phi ,\tau \right)=|0^{\prime }\rangle \langle 0^{\prime }|,\;\;\;\Pi^{\left(1\right)}\left(\alpha ,\phi ,\tau \right)=|1^{\prime }\rangle \langle 1^{\prime }|\} \end{array}$$

with

(28) $$\begin{array}{c} \left(\begin{array}{c} |0^{\prime }\rangle \\ |1^{\prime }\rangle \end{array}\right)=\left(\begin{array}{cc} \cos\alpha e^{i\phi } & \sin\alpha e^{i\tau }\\ -\sin\alpha e^{-i\tau } & \cos\alpha e^{-i\phi } \end{array}\right)\left(\begin{array}{c} |0\rangle \\ |1\rangle \end{array}\right), \end{array}$$

where α∈[0,π/2], ϕ∈[0,2π] and τ∈[0,2π].

If both subsystems are measured by using the parameterized measuring bases (Appendix A), four different outcomes can be obtained as

(29) $$\begin{array}{ccc} p_{bd}^{\left(ij\right)} & = & {\mathrm{Tr}}_{bd}\Omega_{b}^{\left(i\right)}\left(\alpha_{1},\phi_{1},\tau_{1}\right)⊗\Lambda_{d}^{\left(j\right)}\left(\alpha_{2},\phi_{2},\tau_{2}\right)\sigma_{bd} \end{array}$$

Through some tedious deductions, one can obtain

(30) $$\begin{array}{c} \left.\begin{array}{c} p_{bd}^{\left(00\right)}=F\left(w_{2},w_{0},\alpha_{1}\right)\cos^{2}\alpha_{2}+F\left(w_{3},w_{1},\alpha_{1}\right)\sin^{2}\alpha_{2}+\frac{1}{2N}\sqrt{c_{1}c_{2}\left(1-c_{1}\right)\left(1-c_{2}\right)}z_{1}z_{2}\sin2\alpha_{1}\sin2\alpha_{2}\cos\omega ,\\ p_{bd}^{\left(01\right)}=F\left(w_{2},w_{0},\alpha_{1}\right)\sin^{2}\alpha_{2}+F\left(w_{3},w_{1},\alpha_{1}\right)\cos^{2}\alpha_{2}-\frac{1}{2N}\sqrt{c_{1}c_{2}\left(1-c_{1}\right)\left(1-c_{2}\right)}z_{1}z_{2}\sin2\alpha_{1}\sin2\alpha_{2}\cos\omega ,\\ p_{bd}^{\left(10\right)}=F\left(w_{0},w_{2},\alpha_{1}\right)\cos^{2}\alpha_{2}+F\left(w_{1},w_{3},\alpha_{1}\right)\sin^{2}\alpha_{2}-\frac{1}{2N}\sqrt{c_{1}c_{2}\left(1-c_{1}\right)\left(1-c_{2}\right)}z_{1}z_{2}\sin2\alpha_{1}\sin2\alpha_{2}\cos\omega ,\\ p_{bd}^{\left(11\right)}=F\left(w_{0},w_{2},\alpha_{1}\right)\sin^{2}\alpha_{2}+F\left(w_{1},w_{3},\alpha_{1}\right)\cos^{2}\alpha_{2}+\frac{1}{2N}\sqrt{c_{1}c_{2}\left(1-c_{1}\right)\left(1-c_{2}\right)}z_{1}z_{2}\sin2\alpha_{1}\sin2\alpha_{2}\cos\omega , \end{array}\right\} \end{array}$$

where $F\left(s_{1},s_{2},s_{3}\right)\equiv \frac{1}{4}\left(s_{1}\sin^{2}s_{3}+s_{2}\cos^{2}s_{3}\right)$ and $\omega =\phi_{1}+\phi_{2}-\tau_{1}-\tau_{2}$. Combining these bipartite probability distributions, the single-partite probability distributions can be obtained as:

(31) $$\begin{array}{c} \left.\begin{array}{c} p_{b}^{\left(0\right)}=p_{bd}^{\left(00\right)}+p_{bd}^{\left(01\right)}=\frac{1}{4}\left(w_{23}\sin^{2}\alpha_{1}+w_{01}\cos^{2}\alpha_{1}\right),\\ p_{b}^{\left(1\right)}=p_{bd}^{\left(10\right)}+p_{bd}^{\left(11\right)}=\frac{1}{4}\left(w_{01}\sin^{2}\alpha_{1}+w_{23}\cos^{2}\alpha_{1}\right),\\ p_{d}^{\left(0\right)}=p_{bd}^{\left(00\right)}+p_{bd}^{\left(10\right)}=\frac{1}{4}\left(w_{13}\sin^{2}\alpha_{2}+w_{02}\cos^{2}\alpha_{2}\right),\\ p_{d}^{\left(1\right)}=p_{bd}^{\left(01\right)}+p_{bd}^{\left(11\right)}=\frac{1}{4}\left(w_{02}\sin^{2}\alpha_{2}+w_{13}\cos^{2}\alpha_{2}\right). \end{array}\right\} \end{array}$$

Accordingly, the general classical correlation obtained via measure can be expressed as

(32) $$\begin{array}{ccc} C[\sigma_{bd}\left(z_{1},z_{2},c_{1},c_{2}\right)] & = & -\sum_{i=0}^{1}p_{b}^{\left(i\right)}\log_{2}p_{b}^{\left(i\right)}-\sum_{i=0}^{1}p_{d}^{\left(i\right)}\log_{2}p_{d}^{\left(i\right)}+\sum_{i=0}^{1}\sum_{j=0}^{1}p_{bd}^{\left(ij\right)}\log_{2}p_{bd}^{\left(ij\right)}. \end{array}$$

Correspondingly, the usual classical correlation is taken as the maximal one:

(33) $$\begin{array}{c} C_{A}\left[\sigma_{bd}\left(z_{1},z_{2},c_{1},c_{2}\right)\right]=\max_{\left\{\Omega_{b}^{\left(i\right)}⊗\Lambda_{d}^{\left(j\right)}\right\}}C\left[\rho_{bd}\left(z_{1},z_{2},c_{1},c_{2}\right)\right]. \end{array}$$

In order to obtain the maximal value, the extreme points should first be worked out. That is to say, the derivative equations $\partial C\left[\sigma_{bd}\left(z_{1},z_{2},c_{1},c_{2}\right)\right]/\partial \alpha_{1}=\partial C\left[\sigma_{bd}\left(z_{1},z_{2},c_{1},c_{2}\right)\right]/\partial \alpha_{2}=\partial C\left[\sigma_{bd}\left(z_{1},z_{2},c_{1},c_{2}\right)\right]/\partial \omega =0$ should be solved first. However, it is not easy to solve these equations. Fortunately, through observation one can find that the extreme points are $\alpha_{1}=\alpha_{2}=0,\pi /4,\pi /2$ and ω=0. Moreover, through comparing these three points with other points, we find that the value of classical correlation corresponding to this point $\alpha_{1}=\alpha_{2}=0,\pi /4,\pi /2$ and ω=0 is the maximal. Hence, the maximal classical correlation can be expressed

(34) $$\begin{array}{ccc} C_{A}\left[\sigma_{bd}\left(z_{1},z_{2},c_{1},c_{2}\right)\right] & = & \frac{1}{2}[\left(1+\frac{2}{N}\sqrt{c_{1}c_{2}\left(1-c_{1}\right)\left(1-c_{2}\right)}z_{1}z_{2}\right)\log_{2}\left(1+\frac{2}{N}\sqrt{c_{1}c_{2}\left(1-c_{1}\right)\left(1-c_{2}\right)}z_{1}z_{2}\right)\\ & & +\left(1-\frac{2}{N}\sqrt{c_{1}c_{2}\left(1-c_{1}\right)\left(1-c_{2}\right)}z_{1}z_{2}\right)\log_{2}\left(1-\frac{2}{N}\sqrt{c_{1}c_{2}\left(1-c_{1}\right)\left(1-c_{2}\right)}z_{1}z_{2}\right)]. \end{array}$$

Finally, AMID can be obtained as the discrepancy between the total correlation (Equation (15)) and the maximal classical correlation (Equation (34)), i.e.,

(35) $$\begin{array}{ccc} Q_{A}\left[\sigma_{bd}\left(z_{1},z_{2},c_{1},c_{2}\right)\right] & = & I\left[\sigma_{bd}\right]-C_{A}\left[\sigma_{bd}\right]\\ & = & \frac{1}{8}\left[\left(w_{03}+\xi \right)\log_{2}\left(w_{03}+\xi \right)+\left(w_{03}-\xi \right)\log_{2}\left(w_{03}-\xi \right)\right]\\ & - & \frac{1}{4}\left[w_{01}\log_{2}w_{01}+g_{23}\log_{2}w_{23}+w_{02}\log_{2}w_{02}+w_{13}\log_{2}w_{13}\right]\\ & - & \frac{1}{2}[\left(1+\frac{2}{N}\sqrt{c_{1}c_{2}\left(1-c_{1}\right)\left(1-c_{2}\right)}z_{1}z_{2}\right)\log_{2}\left(1+\frac{2}{N}\sqrt{c_{1}c_{2}\left(1-c_{1}\right)\left(1-c_{2}\right)}z_{1}z_{2}\right)\\ & + & \left(1-\frac{2}{N}\sqrt{c_{1}c_{2}\left(1-c_{1}\right)\left(1-c_{2}\right)}z_{1}z_{2}\right)\log_{2}\left(1-\frac{2}{N}\sqrt{c_{1}c_{2}\left(1-c_{1}\right)\left(1-c_{2}\right)}z_{1}z_{2}\right)]\\ & + & \frac{1}{4}\left[w_{1}\log_{2}w_{1}+w_{2}\log_{2}w_{2}-w_{03}\right]+2. \end{array}$$

### 4.3. AMIDs in the Final States $\sigma_{bd}^{\prime \pm }\left(z_{1},z_{2},c_{1},c_{2}\right)$

Similar to Section 4.2, one can find that AMIDs in the two states $\sigma_{bd}^{\prime +}\left(z_{1},z_{2},c_{1},c_{2}\right)$ and $\sigma_{bd}^{\prime -}\left(z_{1},z_{2},c_{1},c_{2}\right)$ are equivalent, and hence one can use $\sigma_{bd}^{\prime }\left(z_{1},z_{2},c_{1},c_{2}\right)$ as the surrogate of $\sigma_{bd}^{\prime \pm }\left(z_{1},z_{2},c_{1},c_{2}\right)$.

Similar to that in Section 4.3, due to the equivalent structure of $\sigma_{bd}^{\prime \pm }\left(z_{1},z_{2},c_{1},c_{2}\right)$ and $\sigma_{bd}^{\pm }\left(z_{1},z_{2},c_{1},c_{2}\right)$, one can obtain AMID $\sigma_{bd}^{\prime }\left(z_{1},z_{2},c_{1},c_{2}\right)$ as

(36) $$\begin{array}{ccc} Q_{A}\left[\sigma_{bd}^{\prime }\left(z_{1},z_{2},c_{1},c_{2}\right)\right] & = & \frac{1}{8}\left[\left(w_{03}^{\prime }+\xi^{\prime }\right)\log_{2}\left(w_{03}^{\prime }+\xi^{\prime }\right)+\left(w_{03}^{\prime }-\xi^{\prime }\right)\log_{2}\left(w_{03}^{\prime }-\xi^{\prime }\right)\right]\\ & - & \frac{1}{4}\left[w_{01}^{\prime }\log_{2}w_{01}^{\prime }+w_{23}^{\prime }\log_{2}w_{23}^{\prime }+w_{02}^{\prime }\log_{2}w_{02}^{\prime }+w_{13}^{\prime }\log_{2}w_{13}^{\prime }\right]\\ & - & \frac{1}{2}[\left(1+\frac{2}{N}\sqrt{c_{1}c_{2}\left(1-c_{1}\right)\left(1-c_{2}\right)}z_{1}z_{2}\right)\log_{2}\left(1+\frac{2}{N}\sqrt{c_{1}c_{2}\left(1-c_{1}\right)\left(1-c_{2}\right)}z_{1}z_{2}\right)\\ & + & \left(1-\frac{2}{N}\sqrt{c_{1}c_{2}\left(1-c_{1}\right)\left(1-c_{2}\right)}z_{1}z_{2}\right)\log_{2}\left(1-\frac{2}{N}\sqrt{c_{1}c_{2}\left(1-c_{1}\right)\left(1-c_{2}\right)}z_{1}z_{2}\right)]\\ & + & \frac{1}{4}\left[w_{1}^{\prime }\log_{2}w_{1}^{\prime }+w_{2}^{\prime }\log_{2}w_{2}^{\prime }-w_{03}^{\prime }\right]+2. \end{array}$$

where all the $w^{\prime }$ quantities related to those in Equation (23) with $W^{,}s$ are replaced by $W^{{}^{\prime },}s$. $W^{,}s$ and $W^{{}^{\prime },}s$ that are listed in Equations (9) and (12), respectively.

## 5. Analyses, Comparisons and Discussion

In the previous two sections, MID and AMID have been respectively utilized to quantify all QCs in the initial and final states. In this section, let us make some analyses, discussions and comparisons.

### 5.1. Features of QCs in the Initail Werner-Like States

The Werner-like state in Equation (2) is comprised of two terms. They are mingled with the weight *z*. One is *I*, a null quantum correlation maximally mixed state. Another state |ψ〉 is an entangled pure state. QC in the |ψ〉 increases with c∈[0,1/2]. Hence, for a fixed *c*, the QC in it is determined. Moreover, the bigger *z* is, the larger weight of |ψ〉. Hence, naturally, a larger QC can be induced by the two mixtures. Particularly, the Werner-like state becomes a Werner state when c=0.5.

To be specific, MID and AMID in the initial Werner-like states have the common features: (i) c=0.5 is a symmetrical point of QC; (ii) for given *c*, MID is an increasing function of *z* and arrives the maximum at z=1; (iii) for a fixed *z*, QC increases with *c* in the region [0,1/2] and reaches maximum at c=1/2.

### 5.2. Monotony Features of MIDs in the Final States

#### 5.2.1. Monotony Features of MIDs in the Final State $\sigma_{bd}\left(z_{1},z_{2},c_{1},c_{2}\right)$

Now let us turn to the monotonic properties of the QCs in the final states (see Equations (23), (24), (34) and (35)). As mentioned, QCs in the final state are determined by four parameters, i.e., $z_{1},z_{2},c_{1},c_{2}$. Obviously, there are two kinds in the four parameters. One kind is $\left(z_{1},z_{2}\right)$ and another is $\left(c_{1},c_{2}\right)$. To find the the monotonic properties is not an easy, because it is quite difficult to judge whether the partial derivatives $\partial Q\left[\sigma_{bd}\left(z_{1},z_{2},c_{1},c_{2}\right)\right]/\partial v_{i}$, v=z,c and i=1,2 are bigger than zero. Hence, we have no choice but to utilize the vast numerical investigations.

**Figure 1:**
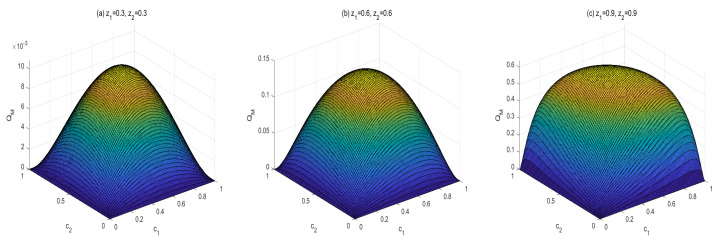
Variation of $Q_{M}\left[\sigma_{bd}\right]$ with $c_{1}$ and $c_{2}$ for three sets of $z_{1}$ and $z_{2}$.

**Figure 2:**
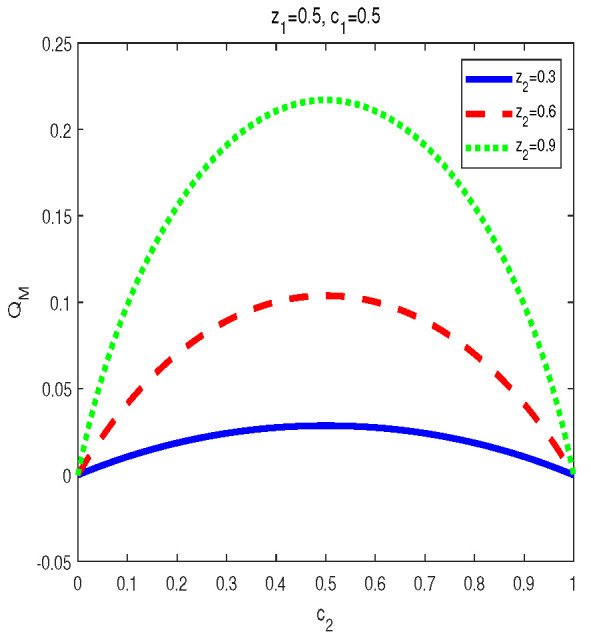
Variation of $Q_{M}\left[\sigma_{bd}\right]$ with $c_{2}$ for $z_{1}=0.5$, $c_{1}=0.5$ and $z_{2}=0.3,0.6,0.9$, respectively.

**Figure 3:**
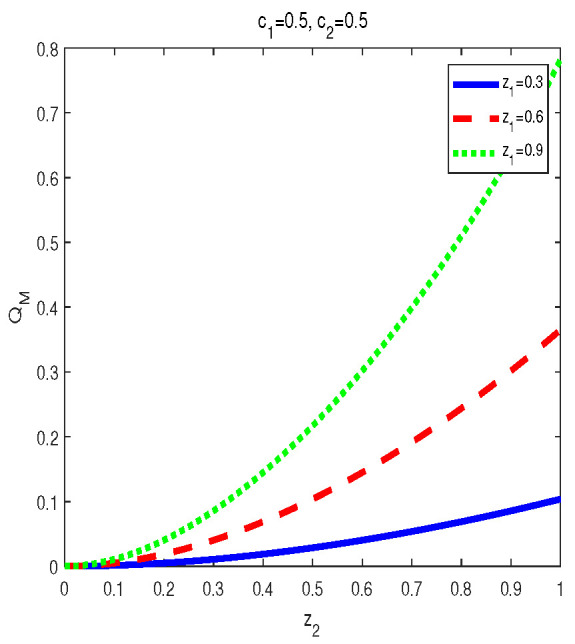
Variation of $Q_{M}\left[\sigma_{bd}\right]$ with $z_{2}$ for $c_{1}=0.5$, $c_{2}=0.5$ and $z_{1}=0.3,0.6,0.9$, respectively.

To obtain the monotony features of MIDs in the final states, vast numerical calculations have been made. Some typical figures are listed in Figure 1, Figure 2 and Figure 3. Through the vast numerical calculations, the following properties have been found:

(1) For given $\left(z_{1},z_{2}\right)$, $Q_{M}\left[\sigma_{bd}\left(z_{1},z_{2},c_{1},c_{2}\right)\right]$ is symmetrical regarding $c_{1}$ and $c_{2}$. To be concrete, $Q_{M}\left[\sigma_{bd}\left(z_{1},z_{2},c_{1},c_{2}\right)\right]$ is increasing in $c_{1}\in \left(0,1/2\right]$ and decreasing in $c_{1}\in \left(1/2,1\right)$. Meanwhile, $Q_{M}\left[\sigma_{bd}\left(z_{1},z_{2},c_{1},c_{2}\right)\right]$ is also increasing in $c_{2}\in \left(0,1/2\right]$ and decreasing in $c_{2}\in \left(1/2,1\right)$. In other words, $Q_{M}\left[\sigma_{bd}\left(z_{1},z_{2},c_{1},c_{2}\right)\right]$ is symmetrical regarding $c_{1}=c_{2}=0.5.$ From Figure 1, one can see that $Q_{M}\left[\sigma_{bd}\left(z_{1},z_{2},c_{1},c_{2}\right)\right]$ is symmetrical regarding $c_{2}=0.5$ and the maximal point occurs at $c_{2}=0.5$. Moreover, the bigger $z_{2}$ is, the bigger the maximal value that can be obtained.

Moreover, a similarity property can be found, i.e., MID in the final state has a similar symmetry property with that in the initial state. That is to say, MIDs in both the two kind states increase in $c_{i}\in \left(0,1/2\right]$ (*i* is 1 or 2) and decrease in $c_{i}\in \left[1/2,1\right)$.

Moreover, One can find that this symmetry property of MID in the final state is similar to that in the initial Werner-like state in Equations (13) and (14). To be concrete, MIDs in both the final state and the initial Werner-like state increase with $c_{i}$ (*i* is 1 or 2) in the region (0,1/2] and decrease with $c_{i}$ in the region [1/2,1). Moreover, there exists an obvious symmetry in $c_{1}=c_{2}=0.5.$ That is to say, taking the final state as example

(37) $$\begin{array}{c} Q_{M}\left[\sigma_{bd}\left(z_{1},z_{2},c_{1}=0.5-\alpha ,c_{2}=0.5-\beta \right)\right]=Q_{M}\left[\sigma_{bd}\left(z_{1},z_{2},c_{1}=0.5+\alpha ,c_{2}=0.5+\beta \right)\right]. \end{array}$$

In Equation (37), α and β are both defined in the region [0,1/2]. This property means that the symmetrical property with $c_{i}$ is unchanged during the QC swapping process. In addition, if $z_{1}$ and $z_{2}$ are bigger, the quantities of QC are larger.

(2) For given $\left(c_{1},c_{2}\right)$, in the final state MID increases with $z_{1}$ or $z_{2}$ in $z_{i}\in \left(0,1\right)$, i=1,2 (see Figure 3). Variations of $Q_{M}\left[\sigma_{bd}\left(z_{1},z_{2},c_{1},c_{2}\right)\right]$ with $z_{2}$ for $c_{1}=0.5,c_{2}=0.5$ and $z_{1}=0.3,0.6,0.9$ are plotted respectively in Figure 3. Obviously, one can see that MID in the final state is an increasing function of $z_{i}$, i=1,2.

#### 5.2.2. Monotony Features of MIDs in the Final State

**Figure 4:**
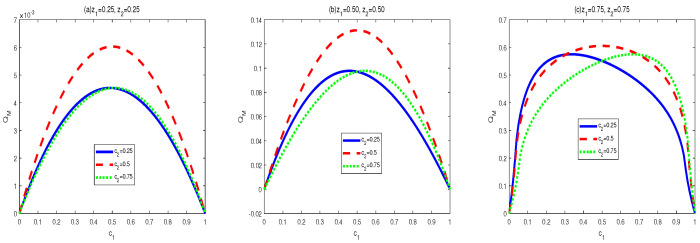
Variation of $Q_{M}\left[\sigma_{bd}^{\prime }\right]$ with $c_{1}$ and $c_{2}$ for three sets of $z_{1}$ and $z_{2}$.

**Figure 5:**
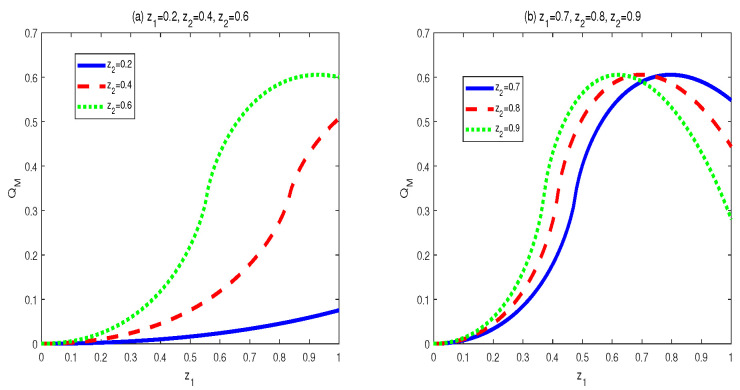
Variation of $Q_{M}\left[\sigma_{bd}^{\prime }\right]$ with $c_{1}=0.5,c_{2}=0.5$ for several sets of $z_{1}$ and $z_{2}$.

As for $\sigma_{bd}^{\prime }\left(z_{1},z_{2},c_{1},c_{2}\right)$, QC quantified by MID is expressed in Equation (24). Some features can be exposed through numerical calculations. See Figure 4 and Figure 5.

(a) For given $c_{2}$ and $\left(z_{1},z_{2}\right)$, $Q_{M}\left[\sigma_{bd}^{\prime }\right]$ first increases then decreases with $c_{1}\in \left(0,1\right]$. The maximal points ($c_{1m}$), i.e., the transition points, vary with parameters. Not only the maximal points and but also the shape of the curves are determined by the value of $c_{2}$. To be specific, the smaller the value of $|c_{2}-0.5|$ is, the bigger maximal value of $Q_{M}\left[\sigma_{bd}^{\prime }\right]$ is. Moreover, for a given set of $\left(z_{1},z_{2}\right)$,

(38) $$\begin{array}{c} Q_{M}\left[\sigma_{bd}\left(z_{1},z_{2},c_{1}=0.5-\alpha ,c_{2}=0.5-\beta \right)\right]=Q_{M}\left[\sigma_{bd}\left(z_{1},z_{2},c_{1}=0.5+\alpha ,c_{2}=0.5+\beta \right)\right] \end{array}$$

where α∈(0,1/2),β∈(0,1/2).

(b) $Q_{M}\left[\sigma_{bd}^{\prime }\right]$ is an increasing function of $z_{1}\in \left[0,1\right]$ within $z_{2}\in \left[0,0.58\right]$. However, when $z_{2}\in \left[0.58,1\right]$, $Q_{M}\left[\rho_{bd}^{\prime }\right]$ first increases then decreases in $z_{1}\in \left[0,1\right]$. Moreover, the bigger $z_{2}\in \left[0.58,1\right]$ is, the smaller of transtion point is.

### 5.3. Monotony Feature of AMIDs in the Final States

#### 5.3.1. Monotony Features of AMIDs in the Final State $\sigma_{bd}\left(z_{1},z_{2},c_{1},c_{2}\right)$

**Figure 6:**
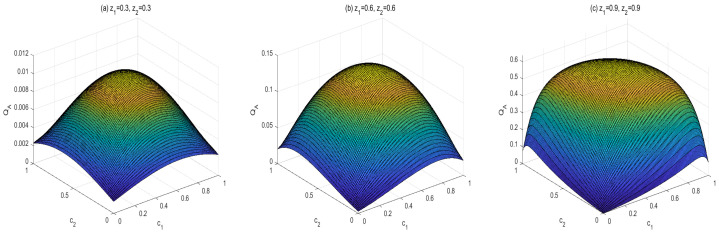
Variation of $Q_{A}\left[\sigma_{bd}\right]$ with $c_{1}$ and $c_{2}$ for three sets of $z_{1}$ and $z_{2}$.

**Figure 7:**
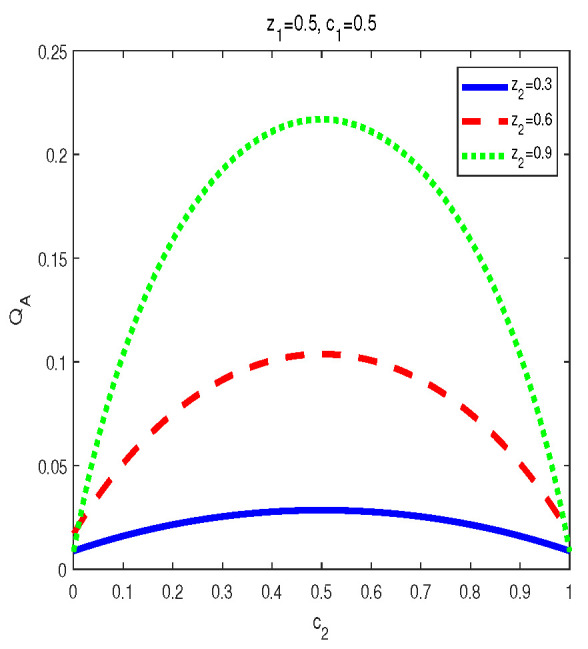
Variation of $Q_{A}\left[\sigma_{bd}\right]$ with $c_{2}$ for $z_{1}=0.5$, $c_{1}=0.5$ and $z_{2}=0.3,0.6,0.9$, respectively.

**Figure 8:**
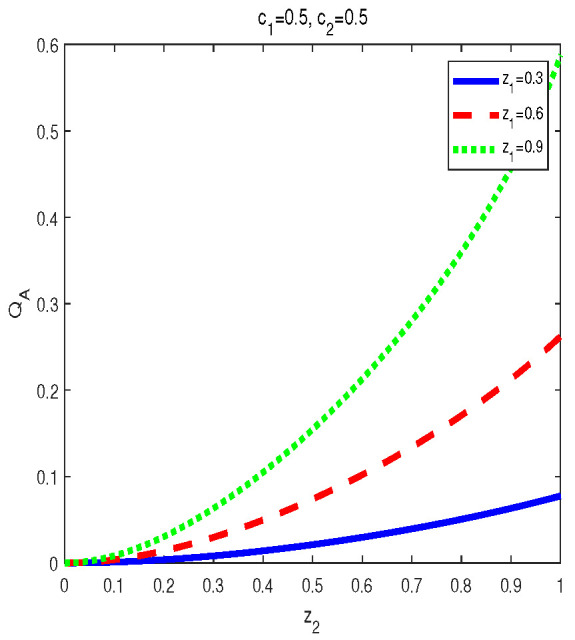
Variation of $Q_{A}\left[\sigma_{bd}\right]$ with $z_{2}$ for $c_{1}=0.5$, $c_{2}=0.5$ and $z_{1}=0.3,0.6,0.9$, respectively.

Now let us look at the monotony features of AMIDs in the final states $\sigma_{bd}\left(z_{1},z_{2},c_{1},c_{2}\right)$. Vast numerical calculations have also been made. Some typical figures are listed in Figure 6, Figure 7 and Figure 8. Through the vast numerical calculations and comparisons, the following properties can be exposed:

(1) $Q_{A}\left[\sigma_{bd}\left(z_{1},z_{2},c_{1},c_{2}\right)\right]$ is symmetrical regarding $c_{1}$ and $c_{2}$ for given $\left(z_{1},z_{2}\right)$, i.e., $Q_{A}$$\left[\sigma_{bd}\left(z_{1},z_{2},c_{1},c_{2}\right)\right]$ is increasing in $c_{i}\in \left(0,1/2\right]$ and decreasing in $c_{i}\in \left(1/2,1\right)$, i=1,2. Moreover, $Q_{A}\left[\sigma_{bd}\left(z_{1},z_{2},c_{1},c_{2}\right)\right]$ is symmetrical regarding $c_{1}=c_{2}=0.5.$ and arrives its maximum at this point. From Figure 2, one can see that $Q_{A}\left[\sigma_{bd}\left(z_{1},z_{2},c_{1},c_{2}\right)\right]$ is symmetrical regarding $c_{2}=0.5$ and the maximal point occurs at $c_{2}=0.5$. Moreover, the bigger $z_{1}$ or $z_{2}$ is, the bigger maximal value can be obtained.

(2) AMID in the final state $\sigma_{bd}\left(z_{1},z_{2},c_{1},c_{2}\right)$ is an increasing function with $z_{i}$ in the region $z_{i}\in \left(0,1\right)$, i=1,2, for given $\left(c_{1},c_{2}\right)$. See Figure 6. In Figure 6, variations of $Q_{A}\left[\sigma_{bd}\left(z_{1},z_{2},c_{1},c_{2}\right)\right]$ with $z_{2}$ for $c_{1}=0.5,c_{2}=0.5$ and $z_{1}=0.3,0.6,0.9$ are plotted respectively. Obviously, one can see that MID in the final state increase with $z_{i}$, i=1,2.

#### 5.3.2. Monotony Features of AMIDs in the Final State $\sigma_{bd}^{\prime }\left(z_{1},z_{2},c_{1},c_{2}\right)$

**Figure 9:**
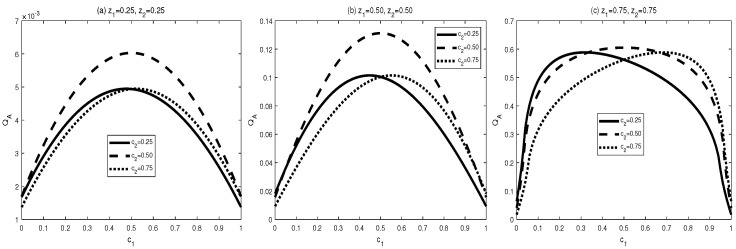
Variation of $Q_{A}\left[\sigma_{bd}^{\prime }\right]$ with $c_{1}$ and $c_{2}$ for three sets of $z_{1}$ and $z_{2}$.

**Figure 10:**
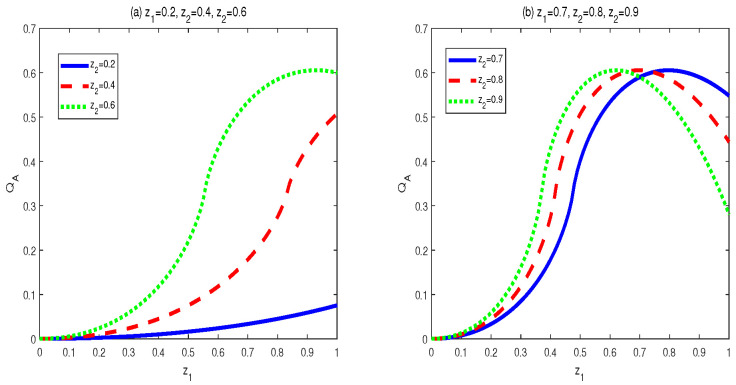
Variation of $Q_{A}\left[\sigma_{bd}^{\prime }\right]$ with $c_{1}$ and $c_{2}$ for three sets of $z_{1}$ and $z_{2}$.

To achieve the properties of AMID in the final state $\sigma_{bd}^{\prime }\left(z_{1},z_{2},c_{1},c_{2}\right)$, we also utilized vast numerical calculations. Some typical figures are listed in Figure 9 and Figure 10. Through the vast numerical calculations and comparisons, the following properties can be exposed.

(a) For given $c_{2}$ and $\left(z_{1},z_{2}\right)$, $Q_{A}\left[\sigma_{bd}^{\prime }\right]$ first increases then decreases with $c_{1}\in \left(0,1\right]$. The maximal points ($c_{1m}$), i.e., the transition points, are changed with different parameters. The maximal points and the shape of the curves are determined by the value of $c_{2}$. Concretely, the closer the value of $c_{1}$ to 0.5 is, the larger maximal value of $Q_{A}\left[\sigma_{bd}^{\prime }\right]$ can be obtained. Moreover, for a given set of $\left(z_{1},z_{2}\right)$,

(39) $$\begin{array}{c} Q_{A}\left[\sigma_{bd}\left(z_{1},z_{2},c_{1}=0.5-\alpha ,c_{2}=0.5-\beta \right)\right]=Q_{M}\left[\sigma_{bd}\left(z_{1},z_{2},c_{1}=0.5+\alpha ,c_{2}=0.5+\beta \right)\right] \end{array}$$

where α∈(0,1/2),β∈(0,1/2).

(b) $Q_{A}\left[\sigma_{bd}^{\prime }\right]$ is an increasing function of $z_{1}\in \left[0,1\right]$ within $z_{2}\in \left[0,0.58\right]$. However, when $z_{2}\in \left[0.58,1\right]$, $Q_{A}\left[\sigma_{bd}^{\prime }\right]$ first increases then decreases in $z_{1}\in \left[0,1\right]$. Moreover, the bigger of $z_{2}\in \left[0.58,1\right]$, the smaller of transtion point is.

### 5.4. Comparisons between MID and AMID in the Final States

In this section, let us make some comparisons between them MID and AMID. From the last sections, through many comparisons, one can obtain the following conclusions:

(i) The properties of MID in the final state $\sigma_{bd}$ are similar to those of AMID in the final state $\sigma_{bd}$. Comparing Figure 1, Figure 2 and Figure 3 with Figure 6, Figure 7 and Figure 8, it is easy to obtain this conclusion. Regardless of the QC—MID or AMID—QCs in the final state $\sigma_{bd}$ are monotonically increasing function of $z_{i}$ (i = 1,2). Additionally, they increase when $c_{i}\in \left[0,1/2\right]$ and symmetrically decrease when $c_{i}\in \left[1/2,1\right]$. These properties are manly due to the middle measurements in Equation (5) during the QC swapping process. In other words, the middle measurements in Equation (5) do not change the QC properties before and after the QC swapping process. To be concrete, the dependent relations of QCs on the parameters ($z_{i}$ and $c_{i}$, i=1,2) in the initial states are retained in the final states.

(ii) The properties of MID in the final state $\sigma_{bd}^{\prime }$ are similar to those of AMID in the final state $\sigma_{bd}^{\prime }$. Comparing Figure 4 and Figure 5 with Figure 9 and Figure 10, one can easily obtain this result. If $c_{2}=0.5$, then QCs (MID or AMID) in the final state $\sigma_{bd}^{\prime }$ are symmetrical regarding $c_{1}=0.5$. Moreover, they are increasing in $c_{1}\in \left[0,1/2\right]$ and decreasing in $c_{1}\in \left[1/2,1\right]$. However, when $c_{2}\neq 0.5$, the symmetry disappears. QCs (MID or AMID) in the final state $\rho_{bd}^{\prime }$ still first increase then decrease, but the transition points are no longer equal to 0.5. As for the dependent relation of QCs (MID or AMID) in the final state $\sigma_{bd}^{\prime }$ on $z_{i}$, some transitions emerge. For example, if $z_{2}\in \left[0.58,1\right]$, QCs (MID or AMID) in the final state $\sigma_{bd}^{\prime }$ first increase then decrease. Obviously, the properties of QCs (MID or AMID) in the final state $\sigma_{bd}^{\prime }$ on the parameters are no longer similar to those in the two initial states. This is mainly due to the middle measurements in Equation (6). That is to say, the middle measurements in Equation (6) changes the properties during the QC swapping process.

(iii) There are some distinct differences between QCs (MID or AMID) in the final state $\sigma_{bd}$ and those in the final state $\sigma_{bd}^{\prime }$. From (i) and (ii), one can see the distict differences. Properties in the QCs (MID or AMID) in the final state $\sigma_{bd}$ are similar to those in the two initial states. However, properties in the QCs (MID or AMID) in the final state $\sigma_{bd}^{\prime }$ are no longer similar to those in the two initial states. The distict differences are mainly due to the two kinds of different measurements in Equations (5) and (6).

(iv) The long-distance QC can be realized indeed. From the above discussions, one can find that QCs in the final states are bigger than zero. That is to say, from the two initial states, i.e., two short-QC owners, one can obtain a final state through QC swapping process. Moreover, the final state is a long-distance QC owner.

In addition, let us look at the influences of entanglement of the initial Werner-like states on the QC swapping in this study. For each of the two Werner-like states in Equations (3) and (4), it is entangled, if and only if $1/2\leq c<1/2(1+\sqrt{\left(z+1)(3z-1\right)}/2z)$ [44]. Hence, one can see that being entangled or not in each one of the initial Werner-like states is determined by this criterion condition. If the criterion condition is not satisfied, there is no entanglement in the Werner-like state, and thus no entanglement swapping. However, from the conclusions discussed above, one can see that the swapping of QC, MID or AMID, is not influenced by the entanglement criterion condition. That is to say, whether entangled or separable in the initial Werner-like states, it does not affect the quantum correlation swapping in this study.

In [42], we discussed quantum correlation swapping between Werner and separable states. In this paper, we discuss the quantum correlation swapping between two Werner-like states. The differences between the two cases can be listed as the following: (1) In the former case, the two initial states are Werner and separable states. The Werner state can be an entanglement state, the while separable state has no entanglement in it. In the latter case, the Werner-like state can be entangled. Moreover, the Werner-like state is a state from the Werner state undergoing local or nonlocal unitary operations. (2) In the former case, there are only two parameters concerned. One is in the Werner state and another is in the separable state. In the latter case, there are four parameters concerned. A Werner-like state has two parameters, one from the original Werner state and another from the unitary operations. (3) The obtained quantities and properties are distinctly different.

Finally, let us make some simple remarks. In this study, we consider a special case of quantum correlation swapping. The two initial states we considered are two Werner-like states. A Werner-like state is determined by two parameters. For convenience, we select the four Bell states with no parameters in them. Hence, in the final states, there are four parameters. In this work, we respectively study the dependence relations on the four parameters.

## 6. Summary

In this paper, the QC swapping with two Werner-like states has been considered. MID and AMID have been utilized to quantify all the QCs in the concerned states. Some distinct features about these obtained QCs have been revealed. Especially, it is found that the monotony features of MID and AMID in the two final states are similar to those in the two initial states, while those in two other final states are not. To be specific, the monotony features of MID and AMID in the two final states in Equation (7) are similar to those in the two initial states. However, the monotony features of MID and AMID in the two final states in Equation (9) are different from those in the two initial states. All these obtained distinct properties will be valuable in the field of quantum information processing.

## Data Availability

Not applicable.

## Appendix A

In order to evaluate AMID in $\sigma_{bd}^{\pm }\left(z_{1},z_{2},c_{1},c_{2}\right)$, a general joint local measurement can be parameterized as $\left\{\Omega_{b}^{\left(i\right)}\left(\alpha_{1},\phi_{1},\tau_{1}\right)⊗\Lambda_{d}^{\left(j\right)}\left(\alpha_{2},\phi_{2},\tau_{2}\right),\;i,j=0,1\right\}$, where $\Omega^{\left(k\right)}$ and $\Lambda^{\left(k\right)}$ take the same forms as that in Equations (27) and (28). If the parameterized measuring bases are used to measure both subsystems, then four different outcomes may occur. Those in Equation (29) are occurrence probabilities of different outcomes. After some tedious deductions, one can obtain those in Equation (30).
